# Amphiregulin in lung diseases: A review

**DOI:** 10.1097/MD.0000000000037292

**Published:** 2024-02-23

**Authors:** Chao Shen, Xiaoping Fan, Yueyan Mao, Junsheng Jiang

**Affiliations:** a Department of Pediatrics, Linping Branch, the Second Affiliated Hospital of Zhejiang University, Hangzhou, China.

**Keywords:** amphiregulin, lung, respiratory system

## Abstract

Amphiregulin is a member of the EGFR family, which is involved in many physiological and pathological processes through its binding with EGFR. Studies have found that amphiregulin plays an important role in the occurrence and development of lung diseases. This paper mainly reviews the structure and function of amphiregulin and focuses on the important role of amphiregulin in lung diseases.

## 1. Introduction

The epidermal growth factor (EGF) family member amphiregulin (AREG) was identified 25 years ago in the supernatant of MCF-7 human breast carcinoma cells treated with phorbol 12-myristate 13-acetate (PMA). It was initially defined as a bifunctional growth factor, capable of inhibiting the proliferation of certain carcinoma cell lines while able to induce that of normal cells such as fibroblasts and keratinocytes.^[1]^ As we all know, AREG is a member of the EGF family, its function is mainly mediated through binding and activating EGFR, a widely expressed tyrosine kinase receptor with 4 types, named ErbB1, ErbB2, ErbB3, and ErbB4. AREG is combined with ErbB1 to trigger the generation of intracellular signals and participate in airway remodeling and inflammation in chronic airway inflammatory diseases.^[2–6]^In this review, we will summarize the essential structural and functional features of AREG and its role in lung disease.

## 2. Struction and function of AREG

In humans, 2 copies of the AREG gene (AREG and AREGB) are found at the EGF family gene cluster on chromosome band 4q13.3, spanning about 10 kb of genomic DNA and located 160 kb apart. The 2 AREG genes are respectively linked to the betacellulin (BTC) genes at the 3 “region and the epiregulin (EREG) and epigenetic (EPGN) genes at the 5” region_._^[7]^ Until now, The relative contribution of each AREG gene copy to the overall level of AREG expression in cells is unknown. Several single nucleotide polymorphisms have been identified in the intergenic region between AREG and AREGB. AREG consists of 6 exons encoding a 1.4 kb mRNA transcript, which can synthesize a transmembrane diregulin precursor composed of 252 amino acids_._^[8]^

Precursor signaling proteins contain multiple domains: A hydrophobic signaling peptide (aa1-20), an n-terminal hydrophilic protein domain with a glycosylation site (aa20-101), a heparin-binding domain with another glycosylation site (aa102-140), an EGF-like domain (aa141-181) with 6 cysteines involved in disulfide bonds, It forms a tricyclic structure, followed by a parapembrane structure (aa182-198) for extracellular dissociation, and also contains a hydrophobic domain (aa199-221) and a cytoplasmic domain (aa222-252) with nuclear localization signals and ubiquitination sites that are associated with AREG endocytosis_._^[9–11]^

According to the different cell microenvironments, the precursor protein contains many glycogenetic motifs and cutting sites and the diregulin can be clipped from different sites. This may lead to the formation of different mature AREG proteins and affect the biological activity of AREG in different cell types. This process is regulated by tumor necrosis fact-A converting enzyme.^[12]^ Transmembrane precursor proteins can activate EGF receptors in a paracrine manner alone or bind to quaternary transmembrane proteins.^[13,14]^

AREG can regulate cell proliferation, apoptosis, and migration of different cell types, including epithelial cells, fibroblasts, and immune cells. Studies have shown that AREG is expressed in a variety of normal tissues, such as the reproductive system, urinary tract system, digestive tract, respiratory system, etc. In recent years, it has also been found that AREG is also expressed in colostrum and serum of healthy people.^[15,16]^ In different conditions, AREG can be expressed in different cells, including congenital lymphoid cells, mast cells, eosinophils, basophils, CD4 + T cells, and CD8 + T cells.^[17]^ Studies have shown^[16]^ that AREG can activate the cascade reaction of receptor cell metabolism, inflammation, and intracellular signaling driven by a variety of induction factors, and promote the growth and survival of normal and transformed cells after binding with EGF receptor.

## 3. AREG and lung disease

Lung disease can be caused by a variety of factors, such as bacterial or viral infections, autoimmune factors, systemic diseases, and lung tumors.^[18]^ The lung diseases caused by these factors are closely related to AREG.

### 3.1. The role of AREG in pulmonary inflammatory diseases

AREG was originally found in the culture medium of human breast cancer cells, therefore the initial research was focused on the field of oncology.^[19–21]^ Recent studies have found that AREG plays an important role in tissue damage repair. After being infected with influenza, Innate lymphoid cells may produce AREG, which is essential for restoring alveolar epithelial cell integrity and tissue repair, moreover, It is also essential for preventing lung damage caused by secondary bacterial infection of Legionella pneumophila.^[22,23]^ Some bacteria, viruses, and related material such as lipopolysaccharides, can regulate AREG expression. When the lungs are stimulated by various inflammations, most immune cells can produce AREG, which may cause an increased expression level of AREG.^[24]^ Some research found that patients infected with novel coronavirus, the level of dimodulin would increase, and it was related to pulmonary fibrosis caused by novel coronavirus pneumonia. The main reason is that during chronic airway inflammation, amphiregulin-producing pathogenic memory T helper-2 cells control the airway fibrotic responses by inducing eosinophils to secrete osteopontin.^[25–27]^

### 3.2. The role of AREG in acute lung injury

Acute lung injury is mainly manifested as hypoxemia and respiratory failure, with high mortality and poor prognosis. The pathogenesis of acute lung injury is complex. At present, the pathological basis of acute lung injury is lung epithelial cell injury caused by excessive activation of inflammatory mediators that infiltrate the lungs and cause lung inflammatory response. Macrophages and neutrophils play an important role in the pathogenesis of acute lung injury. In vitro experiments, it has been confirmed that Areg is expressed in alveolar epithelial cells when they are overstretched, and the Areg gene level is also significantly increased after hyperventilation in isolated lung tissues.^[28,29]^ At the same time, the expression of AREG and EGFR transcripts was significantly increased in posttransplant lung injury, this may indicate that AREG has a role in remodeling the airway. Studies have shown that macrophages produce a high level of AREG in lipopolysaccharide-induced acute lung injury, activate the EGFR-AKT pathway, inhibit inflammatory cell infiltration and inflammatory factor expression, and have a protective effect on lung tissue.^[24,30,31]^ After pretreatment with EGFR blockers and AKT inhibitors, it was found that inhibition of EGFR and AKT almost eliminated the negative regulatory effects of AREG on lung tissue injury and inflammation in acute lung injury, suggesting that the activation of EGFR and AKT is the main mechanism of AREG protective role in lung injury.^[32]^ These findings suggest that AREG plays an important protective role in lung injury and may provide a new target for the treatment of lung injury.

### 3.3. The role of AREG in asthma

Asthma is a heterogeneous disease characterized by chronic airway inflammation and hyperresponsiveness. AREG levels in sputum, airway epithelium, and peripheral blood basophils were significantly increased in patients with asthma.^[33]^ Okumura study found that AREG and EGFR expression levels in nasal mucosa epithelial cells of children with asthma were significantly elevated compared with healthy people.^[34]^ The incidence of asthma is increased in people with rhinovirus infection. Studies have found that AREG can be significantly expressed in human mast cells, basophils, and epithelial cells in rhinovirus-induced asthma, which may be related to the up-regulated expression of AREG in airway epithelial cells, which can cause airway remodeling.^[35,36]^ Airway smooth muscle hyperplasia is one of the main features of tracheal remodeling. Studies have found that AREG can promote airway smooth muscle hyperplasia, and the main mechanism is that bradykinin promotes the secretion of AREG by inducing COX-2 expression, increasing PGE2, and stimulating EP2 and EP4 receptors in smooth muscle cells. Smooth muscle cells produce CXCL8, VEGF and COX-2 induced by AREG. The above studies suggest that AREG is involved in smooth muscle cell proliferation and inflammation in patients with asthma.^[6]^

### 3.4. The role of AREG in bronchopulmonary dysplasia

Bronchopulmonary dysplasia is a chronic lung disease in preterm infants, which occurs mainly due to an imbalance between injury and repair of immature lungs, and stagnation of pulmonary vascular and alveolar growth and development.^[37]^AREG is expressed in both epithelial and mesenchymal cells and regulates proliferation apoptosis, and migration including epithelial, fibroblast, and immune cells.^[1]^ AREG has been shown to promote the proliferation of airway epithelial cells and smooth muscle cells.^[38]^ In a mouse model of bronchopulmonary dysplasia induced by prenatal smoke exposure, lung injury may be associated with AREG-EGFR signaling.^[39]^ Hilliman showed that during mechanical ventilation in preterm lambs, AREG expression in lung tissue was elevated with increasing time and affected alveolar differentiation.^[40]^ In BPD model mice given sildenafil treatment, AREG mRNA expression was reduced and alveolarisation was improved.^[41]^ Upon knockdown of AREG, SP-C, and T1α protein expression was increased, further suggesting that AREG reduction alleviates impaired alveolar transdifferentiation in BPD mice.^[42]^ It has also been observed that increased EGFR and AREG affected the localization of pulmonary myofibroblasts in a lipopolysaccharide-induced rat model of BPD, leading to impaired alveolar development in BPD,^[42]^ all of which suggests that AREG plays an important role in the development of bronchopulmonary dysplasia, and maybe a potential therapeutic target for bronchopulmonary dysplasia.

### 3.5. The role of AREG in lung cancer

AREG expression is increased in a variety of tumors, including colorectal, breast, lung, ovarian, and liver cancers^.[43]^ Studies have shown that AREG can indeed promote carcinogenesis and can act as a pro-cancer factor in most cancers.^[44]^ For example, AREG overexpression provides growth survival signals in lung cancer cells can maintain telomerase activity in epithelial cells, and promote unlimited cell replication.^[45–47]^ Several in vitro experiments and clinical studies have shown that AREG has an important role in the proliferation and survival of transformed cells and can be used as a predictive serological biomarker. For example, AREG can be used as a marker for the sensitivity of gefitinib treatment in non-small cell lung cancer and as a predictive marker for gefitinib resistance,^[48]^ and overexpression of AREG in non-small cell lung cancer results in decreased expression of the pro-apoptotic protein BAX, leading to reduced gefitinib-mediated apoptosis.^[49]^ It was found that inhibition of AREG using Si-RNA in established NSCLC xenograft tumors in rats restored gefitinib drug sensitivity, further demonstrating that AREG-mediated acetylation promotes resistance to gefitinib in NSCLC.^[49]^ In patients unresponsive to gefitinib treatment, increased antiapoptotic activity was associated with AREG overexpression. overexpression of AREG has also been associated with mucinous lung cancer, as inhibition of AREG, as well as the anti-insulin growth factor-1 receptor, using siRNAs has been shown to sensitize cells to gefitinib-induced apoptosis, with a superimposed effect,^[50]^ and all of the above studies suggest that AREG is involved in closely related to lung cancer tumourigenesis, which may provide a broad perspective for clinical application.

## 4. Concluding remarks and future perspectives

As can be inferred from the information summarized herein AREG is indeed a very interesting molecule endowed with multiple and nonredundant biological activities, a large number of studies have confirmed that AREG plays an important role in the development and progression of lung diseases, the precise regulation of the amplitude and duration of these repair mechanisms is essential to avoid tissue damage, and ultimately to avert, thus may provide new therapeutic targets for the clinical treatment of lung diseases. However, the specific mechanism of action of AREG in various lung diseases is still unclear, which also provides a new direction for future research.

## Acknowledgment

The authors thank all of the subjects who participated in this study

## Author contributions

Writing—original draft: Chao Shen.

Conceptualization: Xiaoping Fan.

Formal analysis: Xiaoping Fan.

Resources: Xiaoping Fan, Yueyan Mao.

Methodology: Yueyan Mao.

Project administration: Yueyan Mao.

Software: Yueyan Mao.

Supervision: Junsheng Jiang.
